# Phytochemical Characterization and Biological Activity of *Stachys pinardii* Extracts

**DOI:** 10.1002/cbdv.71812

**Published:** 2026-10-06

**Authors:** Zeyneb Karakus, Cengiz Sarikurkcu, Arzuhan Sihoglu

**Affiliations:** ^1^ Chemical Technology Program Çay Vocational School Afyon Kocatepe University Afyonkarahisar Türkiye; ^2^ Faculty of Pharmacy Afyonkarahisar Health Sciences University Afyonkarahisar Türkiye; ^3^ Department of Pharmacy Services Vocational High School of Health Services Kilis 7 Aralik University Kilis Türkiye

**Keywords:** antioxidant capacity, enzyme inhibition, LC‐ESI‐MS/MS profiling, phenolic constituents, *Stachys pinardii*, ultrasound‐assisted extraction

## Abstract

The study investigated how solvent choice affects the extraction of bioactive phytochemicals from *Stachys pinardii*. Methanol and aqueous extracts of aerial parts were obtained using ultrasound‐assisted extraction and analyzed for phytochemical content, antioxidant capacity, and enzyme inhibition. The methanol extract showed higher total phenolic (52.56 mg GAEs/g) and flavonoid contents (18.19 mg REs/g) than the aqueous extract (15.62 mg GAEs/g and 8.25 mg REs/g). Liquid chromatography–electrospray ionization–tandem mass spectrometry (LC–ESI–MS/MS) analysis identified verbascoside and chlorogenic acid as major compounds in methanol, while aqueous extract was richer in caffeic acid and hydroxycinnamic derivatives. Despite lower phenolic content, the aqueous extract demonstrated stronger antioxidant activity across 2,2‐diphenyl‐1‐picrylhydrazyl (DPPH), 2,2′‐azinobis‐(3‐ethylbenzothiazoline‐6‐sulfonic acid) (ABTS), cupric ion reducing antioxidant capacity (CUPRAC), and ferric reducing antioxidant power (FRAP) assays, with lower IC_50_/EC_50_ values. It also exhibited superior enzyme inhibitory effects, particularly against α‐amylase (2.52 mg/mL) and α‐glucosidase (1.58 mg/mL), although its inhibitory activity remained lower than that of the standard acarbose. Overall, solvent polarity significantly influences both phytochemical composition and biological activity. The aqueous extract, despite its lower total quantified phenolic content, shows promising multifunctional bioactivity. Further studies should focus on bioactivity‐guided fractionation and mechanistic insights into individual compounds.

## Graphical abstract

Ultrasound‐assisted methanol and aqueous extracts of *Stachys pinardii* are comparatively evaluated for phytochemical composition and biological activities. Solvent polarity markedly influences phenolic profiles and bioactivity. Although the methanolic extract contains higher total phenolics, the aqueous extract exhibits superior antioxidant and enzyme inhibitory activities, highlighting its promising multifunctional potential as a natural bioactive source.

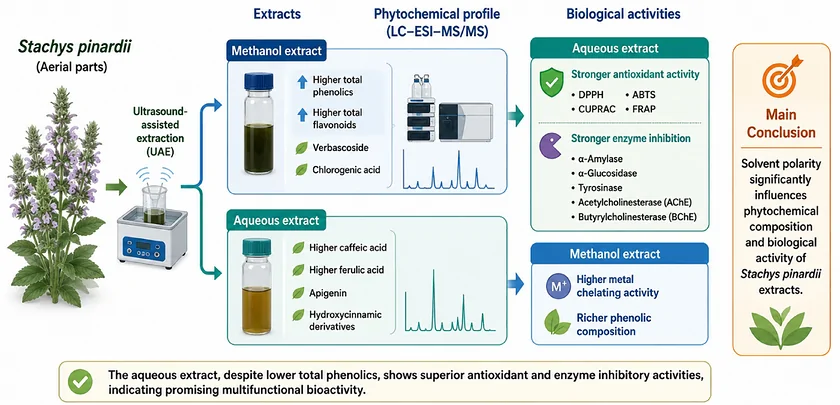

## 1 Introduction

The increasing interest in plant‐derived bioactive compounds is closely associated with their potential roles in mitigating oxidative stress and modulating key enzymes implicated in chronic diseases. Oxidative stress results from an imbalance between the production of reactive oxygen species (ROS) and cellular antioxidant defenses, leading to damage of lipids, proteins, and nucleic acids [1, 2]. This process has been linked to the pathophysiology of various disorders, including metabolic and neurodegenerative diseases, thereby highlighting the importance of identifying effective antioxidant agents [3]. In this context, natural products continue to represent a valuable reservoir of structurally diverse compounds with potential pharmacological relevance [4].

Among medicinally relevant plant groups, the genus *Stachys* L. (Lamiaceae) has attracted considerable attention due to its taxonomic diversity and wide geographical distribution. The genus comprises more than 300 species, many of which are endemic to specific regions and have been traditionally used for various therapeutic purposes [5]. Phytochemical studies have demonstrated that *Stachys* species contain a broad range of secondary metabolites, including phenolic compounds, flavonoids, iridoids, and terpenoids, which are often associated with antioxidant and other biological activities [5, 6]. In particular, phenolic and flavonoid constituents are known to contribute significantly to antioxidant capacity through mechanisms such as free radical scavenging and reducing activity [7, 8].

Despite the increasing body of knowledge on the genus, the distribution of phytochemical and biological data among individual *Stachys* species remains highly uneven. While some taxa have been extensively characterized, others—especially endemic species—have been only partially investigated [5]. *Stachys pinardii* Boiss., an endemic species native to Türkiye, represents one such underexplored taxon. The available literature on this species is limited and primarily focused on its volatile constituents. Ozkan et al. [9] reported the essential oil composition of *S. pinardii*, identifying cedrandiol (25.26%) and caryophyllene dioxide (22.15%) as major components, and also determined the total phenolic content (TPC) of its methanol extract as 600.74 mg gallic acid equivalents (GAE) per 100 g dried herb using the Folin–Ciocalteu method. However, this study did not provide detailed profiling of individual phenolic compounds or a comprehensive evaluation of biological activities.

More recently, Üstüner et al. [10] investigated the essential oil of *S. pinardii* and identified *trans*‐nerolidol (35.7%) and geranyl‐α‐terpinene (17.2%) as the predominant constituents among 33 detected compounds. The same study reported moderate antioxidant activity based on the ABTS radical scavenging assay and moderate α‐glucosidase inhibitory activity, suggesting a certain level of biological potential for the volatile fraction [10]. Nevertheless, this investigation remained limited to essential oil analysis and did not address solvent extracts, which are generally richer in phenolic and flavonoid compounds responsible for pronounced antioxidant effects [8].

The current literature reveals a clear gap in the comprehensive evaluation of *S. pinardii*. Specifically, there is a lack of integrated studies combining detailed phytochemical characterization of nonvolatile extracts with extensive biological activity profiling. In particular, advanced analytical approaches such as LC–ESI–MS/MS‐based quantification of selected phytochemicals, along with the application of multiple antioxidant assays and enzyme inhibition models, have not yet been employed for this species. Such integrated analyses are essential for establishing meaningful relationships between chemical composition and biological activity, which in turn are important for guiding further investigation of their biological relevance [4, 6].

In this context, the present study aims to provide a comprehensive investigation of methanol and aqueous extracts of *S. pinardii*. Total phenolic and flavonoid contents were determined, selected phytochemicals were quantified using LC–ESI–MS/MS, and antioxidant activities were evaluated using multiple complementary assays. In addition, enzyme inhibitory activities were assessed against key target enzymes relevant to metabolic and dermatological disorders. By integrating phytochemical and biological data, this study seeks to expand the current knowledge on *S. pinardii* beyond essential oil‐based findings and to elucidate the relationship between extract composition and bioactivity.

## 2 Results and Discussion

### 2.1 Chemical Composition

The phytochemical profiling of *S. pinardii* extracts revealed a clear solvent‐dependent distribution of phenolic constituents. As shown in Figure 1, the methanol extract exhibited markedly higher total phenolic and total flavonoid contents (TFCs) than the aqueous extract, reaching 52.56 mg GAEs/g extract and 18.19 mg REs/g extract, respectively, whereas the aqueous extract contained 15.62 mg GAEs/g extract and 8.25 mg REs/g extract. These differences were statistically significant according to Student's *t*‐test (*p* < 0.05), indicating that methanol was substantially more effective than water in recovering the overall pool of phenolic and flavonoid compounds from the aerial parts of *S. pinardii* (Figure 1).

**FIGURE 1:**
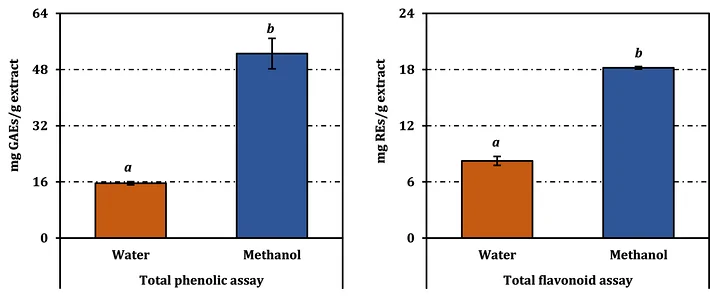
Total flavonoid and phenolic contents of *S. pinardii* extracts. The mean values followed by the same superscripts (a and b) do not differ, according to the Student's *t*‐test at 5% significance level. REs and GAEs represent RE and GAE, respectively.

The targeted LC–ESI–MS/MS analysis further demonstrated that both extracts shared a broadly similar phenolic pattern, although their quantitative composition differed considerably (Table 1). The identities of the four major phenolic constituents (verbascoside, chlorogenic acid, 2,5‐dihydroxybenzoic acid, and protocatechuic acid) were further confirmed by their characteristic LC–ESI–MS/MS product ion spectra and proposed fragmentation pathways, which are presented in Figure S1. These data provide additional structural support for the assignment of the major metabolites identified in *S. pinardii* extracts. Verbascoside was by far the predominant constituent in the methanol extract, reaching 6102 µg/g extract, while it was detected only at a trace level in the aqueous extract (3.68 µg/g extract). Chlorogenic acid also accumulated preferentially in the methanol extract (857 µg/g extract), greatly exceeding its level in the aqueous counterpart (8.00 µg/g extract). Several other compounds were likewise more abundant in methanol, including 2,5‐dihydroxybenzoic acid, protocatechuic acid, pyrocatechol, luteolin 7‐glucoside, rosmarinic acid, and quercetin. In addition, gallic acid, hyperoside, vanillin, taxifolin, and apigenin 7‐glucoside were detected exclusively in the methanol extract.

**TABLE 1:** Concentration (µg/g extract) of selected phenolic compounds in *S. pinardii* extracts.

Compounds	Methanol	Aqueous
Verbascoside	6102 ± 7^a^	3.68 ± 0.07^b^
Chlorogenic acid	857 ± 2^a^	8.00 ± 0.06^b^
2,5‐Dihydroxybenzoic acid	381 ± 4^a^	296 ± 8^b^
Protocatechuic acid	371 ± 2^a^	294 ± 5^b^
4‐Hydroxybenzoic acid	81.6 ± 0.3^b^	96.4 ± 0.7^a^
3‐Hydroxybenzoic acid	74.6 ± 0.2^b^	96.3 ± 0.2^a^
Pyrocatechol	67.2 ± 0.7^a^	49.9 ± 0.4^b^
Apigenin	55.7 ± 0.1^b^	147 ± 4^a^
Gallic acid	39.5 ± 0.3	nd
Syringic acid	31.1 ± 0.1^b^	79.5 ± 0.2^a^
Hyperoside	27.3 ± 0.1	nd
*p*‐Coumaric acid	26.3 ± 0.1^b^	79.9 ± 0.6^a^
Pinoresinol	15.8 ± 0.2^b^	73.2 ± 0.9^a^
Caffeic acid	13.1 ± 0.1^b^	321 ± 1^a^
Luteolin 7‐glucoside	11.5 ± 0.1^a^	0.98 ± 0.01^b^
Ferulic acid	10.1 ± 0.1^b^	133 ± 1^a^
Hesperidin	9.44 ± 0.03^b^	13.6 ± 0.4^a^
Vanillin	8.61 ± 0.01	nd
Luteolin	7.10 ± 0.12^b^	13.9 ± 0.1^a^
Rosmarinic acid	6.79 ± 0.01^a^	3.70 ± 0.07^b^
Taxifolin	5.56 ± 0.08	nd
Apigenin 7‐glucoside	2.23 ± 0.06	nd
Quercetin	2.12 ± 0.03^a^	0.98 ± 0.03^b^
(−)‐Epicatechin	nd	nd
(+)‐Catechin	nd	nd
2‐Hydroxycinnamic acid	nd	nd
3,4‐Dihydroxyphenylacetic acid	nd	nd
Eriodictyol	nd	nd
Kaempferol	nd	nd
Sinapic acid	nd	nd

The mean values followed by the same superscripts (a and b) within a row do not differ, according to the Student's *t*‐test at 5% significance level. nd: Not detected.

By contrast, the aqueous extract was characterized by higher concentrations of several simple phenolic acids and flavonoids. Notably, caffeic acid was especially enriched in the aqueous extract (321 µg/g extract vs. 13.1 µg/g extract in methanol), together with apigenin (147 vs. 55.7 µg/g extract), ferulic acid (133 vs. 10.1 µg/g extract), 4‐hydroxybenzoic acid (96.4 vs. 81.6 µg/g extract), 3‐hydroxybenzoic acid (96.3 vs. 74.6 µg/g extract), *p*‐coumaric acid (79.9 vs. 26.3 µg/g extract), syringic acid (79.5 vs. 31.1 µg/g extract), and pinoresinol (73.2 vs. 15.8 µg/g extract). Hesperidin and luteolin were also present at significantly higher levels in the aqueous extract, although the differences were less pronounced.

The quantitative data indicate that the methanol extract was dominated by a smaller number of highly concentrated phenolics, particularly verbascoside and chlorogenic acid, which likely contributed to its markedly higher total phenolic and flavonoid values. In contrast, the aqueous extract displayed a comparatively lower total phenolic burden but was relatively richer in selected hydroxybenzoic and hydroxycinnamic acid derivatives as well as certain flavonoids. No detectable amounts of (−)‐epicatechin, (+)‐catechin, 2‐hydroxycinnamic acid, 3,4‐dihydroxyphenylacetic acid, eriodictyol, kaempferol, or sinapic acid were found in either extract.

The present investigation provides the first comprehensive characterization of the nonvolatile phytochemical profile of *S. pinardii*, thereby addressing a clear gap in the existing literature, which has so far been limited exclusively to essential oil analyses of this species. Previous studies have focused on volatile constituents, reporting sesquiterpene‐rich profiles dominated by compounds such as *trans*‐nerolidol, geranyl‐ α‐terpinene, α‐curcumene, and caryophyllene derivatives [10, 11]. While these findings have contributed valuable insights into the aromatic fraction of the species, they provide no information on its phenolic and flavonoid composition, which are often more directly associated with a wide range of biological activities. In this context, the current study represents the first detailed report on the nonvolatile chemical composition of *S. pinardii*, significantly expanding the phytochemical knowledge of the species.

The results clearly demonstrate a pronounced solvent‐dependent extraction pattern, with methanol proving more effective than water in recovering phenolic and flavonoid constituents. This can be attributed to the intermediate polarity of methanol, which enables the extraction of a broader spectrum of phenolic metabolites, including both moderately polar flavonoids and phenolic acids, while also facilitating solvent penetration into plant tissues and disrupting interactions between phenolic compounds and cell wall components. Consequently, compounds such as verbascoside and chlorogenic acid were extracted much more efficiently with methanol, contributing substantially to the higher total phenolic and flavonoid contents observed in this extract. This observation is consistent with numerous reports on other *Stachys* taxa, where methanol has been identified as a more efficient solvent for extracting polyphenolic compounds due to its intermediate polarity and ability to solubilize both moderately polar and nonpolar phenolics [12, 13]. For instance, in *Stachys tmolea*, the methanol extract was similarly enriched in key phenolics such as verbascoside, chlorogenic acid, and apigenin derivatives [14]. The dominance of these compounds in the methanol extract of *S. pinardii* aligns well with this broader chemotaxonomic trend within the genus.

Particularly noteworthy is the predominance of verbascoside and chlorogenic acid in the methanol extract, both of which are widely recognized as characteristic markers in *Stachys* species. Similar compositional patterns have been reported in *Stachys cretica* subsp. *kutahyensis* and *S. tmolea*, where these compounds were identified as major constituents through LC–ESI–MS/MS analyses [13, 14]. The recurrence of these phenolics across multiple *Stachys* taxa suggests a conserved biosynthetic capacity within the genus.

In contrast, the aqueous extract of *S. pinardii* exhibited a distinct compositional profile, characterized by relatively higher levels of simple hydroxybenzoic and hydroxycinnamic acids, as well as certain flavonoids. It is important to note that the direction of solvent efficiency is not universally consistent across all *Stachys* species. For example, in *S. cretica* subsp. *anatolica*, aqueous extracts were reported to contain higher total phenolic and flavonoid contents than methanol extracts [15], highlighting the influence of species‐specific matrix effects and compound distribution.

Comparative analysis with other *Stachys* species further supports the chemical richness of *S. pinardii*. Studies on *Stachys lavandulifolia* have revealed a diverse phenolic composition including luteolin, caffeic acid, and quercetin as major constituents [16], while *Stachys macrantha* has been reported to contain significant levels of kaempferol and quercetin [17]. Although direct quantitative comparisons are limited by differences in analytical methods and extraction protocols, the presence of structurally related phenolics across these species underscores a common phytochemical framework within the genus.

Collectively, these findings expand the current phytochemical knowledge of *S. pinardii* and provide a useful basis for future studies aimed at correlating individual phenolic constituents with biological activities, as well as for the identification of potential chemotaxonomic markers within the genus.

### 2.2 Antioxidant Activity

The antioxidant capacities of *S. pinardii* extracts were comparatively evaluated using multiple in vitro assays, and the results are summarized in Table 2. In general, the aqueous extract demonstrated superior radical scavenging and reducing potential compared to the methanol extract, whereas the latter exhibited a comparatively stronger metal chelating capacity.

**TABLE 2:** Antioxidant activities of *S. pinardii* extracts.

Assays	Methanol	Aqueous	Trolox	EDTA
DPPH radical scavenging (IC_50_: mg/mL)	10.30 ± 0.22^c^	4.50 ± 0.04^b^	0.20 ± 0.02^a^	—
ABTS radical cation scavenging (IC_50_: mg/mL)	3.75 ± 0.06^c^	2.55 ± 0.04^b^	0.19 ± 0.01^a^	—
Ferrous ion chelating (IC_50_: mg/mL)	1.08 ± 0.01^b^	1.55 ± 0.004^c^	—	0.02 ± 0.002^a^
Phosphomolybdenum (EC_50_: mg/mL)	1.61 ± 0.01^c^	1.03 ± 0.001^b^	0.40 ± 0.01^a^	—
CUPRAC reducing power (EC_50_: mg/mL)	6.04 ± 0.31^c^	1.03 ± 0.03^b^	0.18 ± 0.02^a^	—
FRAP reducing power (EC_50_: mg/mL)	1.38 ± 0.12^c^	0.56 ± 0.01^b^	0.043 ± 0.004^a^	—

The mean values followed by the same superscripts (a, b, and c) within the same row do not differ, according to the Tukey's honestly significant difference post hoc test at 5% significance level. EC_50_ (mg/mL), effective concentration at which the absorbance was 0.5 for reducing power and phosphomolybdenum assays. IC_50_ (mg/mL), inhibition concentration at which 50% of the DPPH and ABTS radicals were scavenged and the formation of the ferrous ion–ferrozine complex was inhibited. EDTA, ethylenediaminetetraacetic acid (disodium salt). “—”, not determined.

In the radical scavenging assays, the aqueous extract showed significantly lower IC_50_ values than the methanol extract, indicating higher activity. Specifically, the IC_50_ values were 4.50 mg/mL (aqueous) and 10.30 mg/mL (methanol) for DPPH, and 2.55 mg/mL (aqueous) and 3.75 mg/mL (methanol) for ABTS (Table 2). Despite this, both extracts were markedly less potent than the reference antioxidant Trolox. A similar trend was observed in the total antioxidant capacity assay, where the phosphomolybdenum EC_50_ value of the aqueous extract (1.03 mg/mL) was notably lower than that of the methanol extract (1.61 mg/mL), again indicating higher efficacy.

The reducing power assays further supported the enhanced antioxidant performance of the aqueous extract. In the CUPRAC assay, the EC_50_ value of the aqueous extract (1.03 mg/mL) was substantially lower than that of the methanol extract (6.04 mg/mL). Likewise, in the FRAP assay, the aqueous extract (0.56 mg/mL) exhibited stronger reducing capacity than the methanol extract (1.38 mg/mL). Nevertheless, both extracts remained less active than Trolox across these assays.

In contrast, the ferrous ion chelating assay revealed a different pattern. The methanol extract demonstrated a significantly stronger chelating ability (IC_50_: 1.08 mg/mL) compared to the aqueous extract (IC_50_: 1.55 mg/mL), although both were considerably weaker than the standard chelator EDTA.

Statistical analysis confirmed that all observed differences between the extracts were significant (*p* < 0.05), as indicated by distinct superscript letters within each assay. Additionally, antioxidant capacities expressed as Trolox or EDTA equivalents are presented in Figure 2, providing a complementary representation of the findings.

**FIGURE 2:**
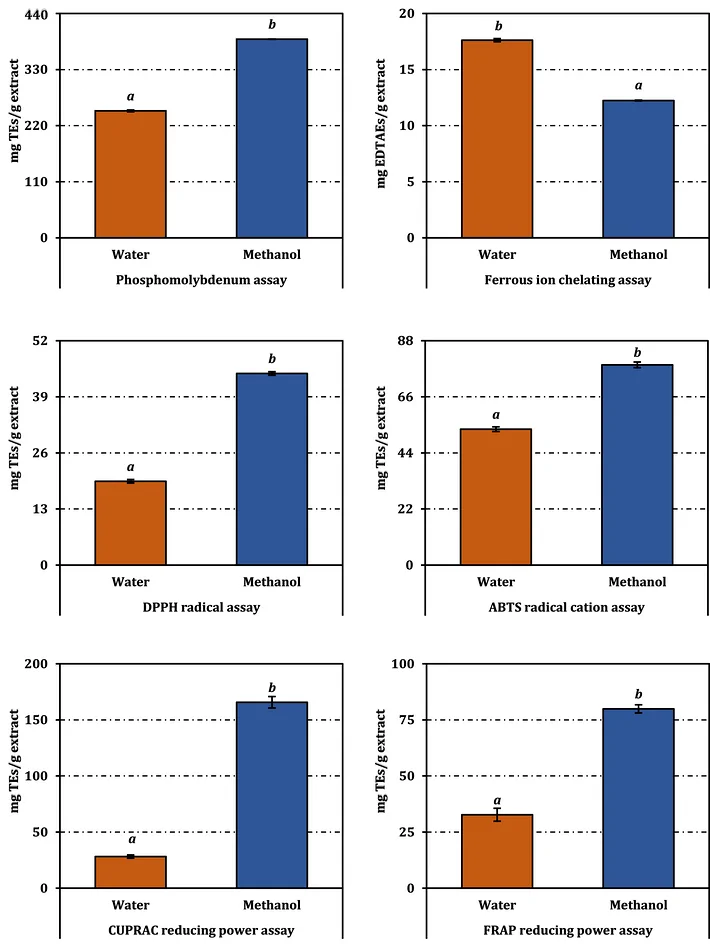
Antioxidant activity of *S. pinardii* extracts. The mean values followed by the same superscripts (a and b) do not differ, according to the Student's *t*‐test at 5% significance level. TEs and EDTAEs denote Trolox and ethylenediaminetetraacetic acid (disodium salt) equivalents, respectively.

Previous studies on *S. pinardii* have mainly focused on the antioxidant activity of its essential oil, reporting only moderate radical scavenging effects [10]. The present results broaden the available knowledge by demonstrating that polar extracts also possess measurable antioxidant activity, although with clear solvent‐dependent differences. More importantly, the observed differences between methanol and aqueous extracts provide an opportunity to better understand how extraction solvent influences the recovery of compounds contributing to different antioxidant mechanisms. While methanol preferentially extracted higher amounts of total phenolics and characteristic constituents such as verbascoside and chlorogenic acid, the aqueous extract consistently showed stronger radical scavenging and reducing activities. This finding indicates that antioxidant performance depends not only on the quantity of phenolics but also on their qualitative composition, molecular interactions, and the presence of other water‐soluble antioxidant constituents.

In addition, different antioxidant assays rely on distinct reaction mechanisms, including hydrogen atom transfer (HAT), single electron transfer (SET), or metal ion coordination [18, 19]. Therefore, compounds exhibiting moderate activity in one assay may perform differently in another depending on their redox potential, hydroxyl substitution pattern, and metal‐binding capacity. The superior performance of the aqueous extract in DPPH, ABTS, CUPRAC, and FRAP assays may therefore reflect enrichment in highly reactive hydrophilic phenolics and other polar reducing constituents, whereas the stronger metal‐chelating activity of the methanol extract may be associated with phenolics possessing more favorable coordination sites for Fe^2^^+^ ions. This mechanistic interpretation is consistent with previous reports emphasizing that different classes of phenolic metabolites contribute selectively to distinct antioxidant pathways rather than exerting uniform effects across all antioxidant assays [19].

A key outcome of this study is the solvent‐dependent differentiation in antioxidant behavior. At first glance, this finding appears unexpected because the methanol extract contained substantially higher total phenolic and flavonoid contents and was particularly enriched in verbascoside and chlorogenic acid, both of which are well‐recognized antioxidants with potent radical‐scavenging and reducing capacities. Nevertheless, antioxidant activity of complex plant extracts is not determined solely by the abundance of one or two major compounds. Instead, it reflects the combined contribution of the entire phytochemical matrix, including compound diversity and relative proportions of the constituents [20]. Therefore, despite its lower TPC, the aqueous extract may contain a more favorable combination of highly active hydrophilic phenolics, unidentified water‐soluble antioxidants, and other polar metabolites that may contribute to the observed activity. Consequently, the exceptionally high levels of verbascoside and chlorogenic acid in the methanol extract did not necessarily translate into superior antioxidant activity, indicating that qualitative phytochemical composition may be more relevant than the absolute abundance of these individual compounds. This observation is consistent with previous reports demonstrating that antioxidant performance can reflect the combined contribution of multiple constituents within complex phytochemical mixtures rather than by the concentration of dominant phenolics alone [20]. Comparable patterns have also been reported in several *Stachys* taxa, where aqueous or decoction‐based extracts exhibit strong radical scavenging and reducing capacities due to their enrichment in hydrophilic phenolics [13, 21]. Similarly, studies on *Stachys annua* demonstrated that both aqueous and methanol extracts possess considerable antioxidant potential, although the relative dominance of one solvent over another varies depending on phytochemical composition [22].

Interestingly, the relatively stronger metal chelating activity of the methanol extract indicates a distinct phytochemical profile enriched in compounds capable of coordinating transition metals. This divergence between radical scavenging/reducing power and metal chelation is not uncommon and has been previously noted in *Stachys* species, where different classes of secondary metabolites contribute selectively to antioxidant sub‐mechanisms [23, 24].

When positioned within the broader *Stachys* literature, the current findings reveal both consistencies and deviations. For instance, several studies report methanol extracts as the most active fractions, particularly in reducing power assays such as CUPRAC and FRAP [24, 25, 26]. However, the dominance of the aqueous extract observed here suggests that *S. pinardii* may possess a phytochemical composition skewed toward more polar antioxidant constituents, a pattern also observed in *S. cretica* subsp. *kutahyensis*, where aqueous extracts showed enhanced activity in certain assays [13]. Conversely, species such as *S. lavandulifolia* and *Stachys antalyensis* often demonstrate stronger activity in alcoholic extracts, highlighting interspecific variability in metabolite distribution [25].

These comparisons indicate that antioxidant behavior within the genus is highly species‐specific and strongly influenced by extraction solvent, reflecting differences in phenolic composition and molecular polarity. The present study contributes novel evidence that *S. pinardii* follows a pattern where polar extracts are particularly effective in electron‐donating and radical‐neutralizing processes, while less polar fractions may preferentially contribute to metal chelation.

In conclusion, beyond establishing baseline antioxidant data for *S. pinardii*, this work underscores the importance of solvent selection in accurately capturing the bioactive profile of *Stachys* species and provides a foundation for future phytochemical and mechanistic investigations.

### 2.3 Enzyme Inhibition Activity

The enzyme inhibitory properties of the methanol and aqueous extracts of *S. pinardii* were comparatively evaluated against acetylcholinesterase (AChE), butyrylcholinesterase (BChE), tyrosinase, α‐amylase, and α‐glucosidase, and the results are summarized in Table 3. For consistency, only IC_50_ values are discussed herein, while the corresponding activities expressed as positive control equivalents are presented in Figure 3.

**TABLE 3:** Enzyme inhibition activity of *S. pinardii* extracts.

Samples	Methanol	Aqueous	Galanthamine	Kojic acid	Acarbose
AChE inhibition (IC_50_: mg/mL)	1.52 ± 0.01^c^	1.28 ± 0.10^b^	0.0028 ± 0.0002^a^	—	—
BChE inhibition (IC_50_: mg/mL)	3.22 ± 0.06^c^	2.65 ± 0.22^b^	0.0032 ± 0.0002^a^	—	—
Tyrosinase inhibition (IC_50_: mg/mL)	3.88 ± 0.03^c^	1.12 ± 0.01^b^	—	0.076 ± 0.004^a^	—
α‐Amylase inhibition (IC_50_: mg/mL)	22.97 ± 0.68^b^	2.52 ± 0.01^a^	—	—	0.91 ± 0.02^a^
α‐Glucosidase inhibition (IC_50_: mg/mL)	4.89 ± 0.17^b^	1.58 ± 0.03^a^	—	—	1.25 ± 0.04^a^

Values indicated by the same superscripts (a–c) within the same row do not differ significantly according to Tukey's honestly significant difference (HSD) post hoc test at 5% significance level. IC_50_ (mg/mL), inhibition concentration at which 50% of the enzymes were inhibited.

**FIGURE 3:**
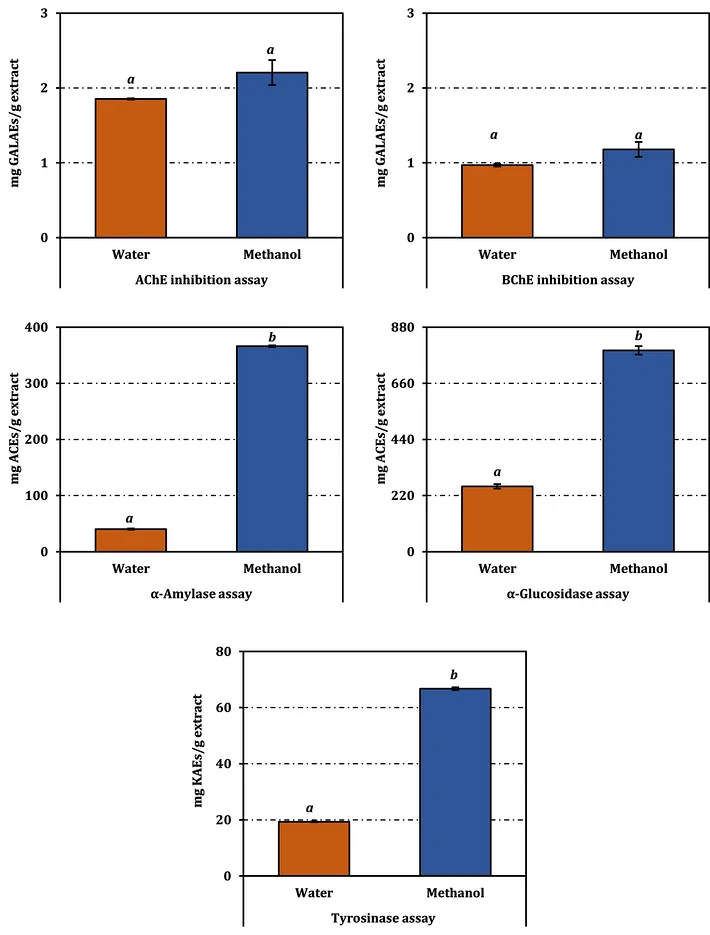
Enzyme inhibition activity of *S. pinardii* extracts. Values are presented as mean ± SD (*n* = 3). Different letters indicate statistically significant differences according to Student's *t*‐test (*p* < 0.05). ACEs, GALAEs, and KAEs denote acarbose equivalents, galanthamine equivalents, and kojic acid equivalents, respectively.

Both extracts exhibited moderate inhibitory activity against cholinesterases, with the aqueous extract demonstrating relatively stronger effects than the methanol extract. Specifically, AChE inhibition was observed with IC_50_ values of 1.28 mg/mL for the aqueous extract and 1.52 mg/mL for the methanol extract, while BChE inhibition followed a similar pattern, yielding IC_50_ values of 2.65 and 3.22 mg/mL, respectively (Table 3). Despite this trend, both extracts were markedly less potent than the reference compound galanthamine.

In terms of tyrosinase inhibition, the aqueous extract showed a substantially higher activity (IC_50_: 1.12 mg/mL) compared to the methanol extract (IC_50_: 3.88 mg/mL), although both remained considerably weaker than kojic acid. A pronounced difference between the extracts was also evident in carbohydrate‐hydrolyzing enzyme inhibition. The aqueous extract exhibited stronger α‐amylase inhibitory activity (IC_50_: 2.52 mg/mL) than the methanol extract (IC_50_: 22.97 mg/mL), although both extracts remained less potent than the reference inhibitor acarbose (IC_50_: 0.91 mg/mL). A similar trend was observed for α‐glucosidase inhibition, where the aqueous extract (IC_50_: 1.58 mg/mL) outperformed the methanol extract (IC_50_: 4.89 mg/mL), while its inhibitory activity remained slightly weaker than that of acarbose (IC_50_: 1.25 mg/mL).

The inhibitory profile clearly indicates that the aqueous extract consistently exhibits higher enzyme inhibition capacity than the methanol extract across all tested targets, with statistically significant differences confirmed by Tukey's post hoc test (*p* < 0.05). The graphical representation of these activities in terms of standard equivalents can be consulted in Figure 3 for complementary interpretation.

The enzyme inhibitory profile of *S. pinardii* extracts provides a noteworthy contribution to the existing body of knowledge, particularly given the scarcity of prior investigations on this species beyond its essential oil. To date, only the α‐glucosidase inhibitory activity of the essential oil of *S. pinardii* has been reported, demonstrating moderate activity [10]. In contrast, there has been no available information regarding the enzyme inhibitory potential of nonvolatile extracts of this species. Therefore, the present findings represent the first comprehensive report on the inhibitory effects of polar extracts of *S. pinardii* against a panel of clinically relevant enzymes, including cholinesterases, tyrosinase, and carbohydrate‐hydrolyzing enzymes, thereby addressing a clear gap in the literature.

A key outcome of this study is the consistently higher inhibitory performance of the aqueous extract compared to the methanol extract across all tested enzymes. This observation is particularly interesting when contextualized within the broader *Stachys* literature, where solvent‐dependent variations in bioactivity are frequently reported but not always consistent. For instance, Kirkan [12] demonstrated that methanol extracts of *S. cretica* subsp. *vacillans* exhibited superior inhibition of α‐amylase and tyrosinase compared to aqueous extracts, highlighting a contrasting solvent–activity relationship relative to the present findings. Such discrepancies may be attributed to interspecific differences in phytochemical composition, as well as the differential solubility of active constituents.

The relatively modest inhibition of AChE and BChE observed in this study aligns with previous reports on other *Stachys* species. For example, *S. annua* extracts were shown to possess weak cholinesterase inhibitory effects despite exhibiting stronger activity against carbohydrate‐hydrolyzing enzymes [22]. Similarly, in *S. lavandulifolia*, notable cholinesterase inhibition was primarily associated with less polar extracts such as *n*‐hexane and dichloromethane fractions [27], suggesting that nonpolar constituents may play a more prominent role in cholinesterase inhibition within the genus. This may partly explain the limited activity observed in the polar extracts of *S. pinardii*.

In contrast, the pronounced inhibitory effects of the aqueous extract against α‐amylase and α‐glucosidase are consistent with trends reported in several *Stachys* taxa. Both methanol and aqueous extracts of *S. annua* have been shown to exhibit strong inhibition of these enzymes [22], while *Stachys palustris* extracts demonstrated significant activity depending on plant part and phenolic content [28]. Moreover, essential oils of *Stachys viticina* also exhibited inhibitory effects against these enzymes, albeit with variable potency [29]. Collectively, these findings provide preliminary in vitro evidence that *Stachys* species contain constituents capable of inhibiting carbohydrate‐hydrolyzing enzymes. However, considering that most inhibitory activities were observed in the mg/mL range, these results should be interpreted cautiously and regarded as an initial basis for further phytochemical, mechanistic, and in vivo investigations rather than evidence of direct therapeutic applicability.

The observed tyrosinase inhibition, particularly in the aqueous extract, further reinforces the functional versatility of *Stachys* species. Previous studies have demonstrated that different solvent fractions of *Stachys byzantina* and *S. lavandulifolia* possess measurable anti‐tyrosinase activity [27, 30]. However, similar to cholinesterase inhibition, stronger activity is often associated with less polar or semipolar extracts, suggesting that distinct classes of compounds may be responsible for enzyme‐specific effects.

The enzyme inhibition profile of *S. pinardii* highlights a differential activity pattern that is strongly influenced by extraction solvent and likely reflects the underlying phytochemical distribution. The superior performance of the aqueous extract, particularly against carbohydrate‐hydrolyzing enzymes, is unlikely to be explained solely by the quantified phenolic profile. Instead, the observed activity may result from the combined effects of unidentified polar constituents, multiple metabolites, matrix‐related effects in the bioassays, and potentially water‐soluble non‐phenolic compounds. Therefore, the biological activity of the aqueous extract should be interpreted as the outcome of a complex phytochemical matrix rather than being attributed exclusively to the quantified phenolic constituents.

In conclusion, this study not only provides the first evidence of enzyme inhibitory activities of nonvolatile extracts of *S. pinardii*, but also situates these findings within the broader phytochemical and pharmacological landscape of the *Stachys* genus. The results underscore the importance of solvent selection in bioactivity studies and suggest that *S. pinardii* may serve as a promising candidate for further investigation to clarify the biological relevance of the observed in vitro enzyme inhibitory activities and to determine their potential significance through further mechanistic and in vivo studies.

## 3 Conclusions

This study demonstrates that the biological performance of *S. pinardii* extracts is governed more by qualitative compositional features than by the overall abundance of phenolics. The observed divergence between extraction efficiency and bioactivity emphasizes that higher total phenolic or flavonoid content does not necessarily translate into stronger functional effects, underscoring the importance of compound‐specific contributions and the overall phytochemical composition of the extracts.

The findings collectively point to a clear selectivity in how different solvent systems shape the in vitro bioactivity profile of the plant. In this context, water‐based extraction emerges as a particularly relevant approach for obtaining extracts with measurable in vitro bioactivity, suggesting that more sustainable and biologically effective extraction strategies can be aligned. This aspect is especially valuable when considering potential future applications, subject to further validation, where solvent safety and regulatory compatibility are critical factors.

Moreover, the observed enzyme inhibition patterns constitute preliminary in vitro evidence of the biological potential of *S. pinardii*. Although the aqueous extract consistently exhibited stronger inhibitory activity than the methanol extract, most IC_50_ values remained in the mg/mL range and were generally weaker than those of the corresponding reference inhibitors. Therefore, these findings should be considered an initial indication of bioactivity that warrants further phytochemical characterization, bioactivity‐guided fractionation, mechanistic studies, and in vivo validation before any therapeutic relevance can be inferred.

In a broader context, these results reinforce the need to move beyond bulk compositional metrics and adopt more integrative evaluation strategies that link phytochemical profiling with biological outcomes. Future work focusing on bioactivity‐guided isolation, interaction studies among key constituents, and in vivo validation will be essential to establish the broader biological relevance of these preliminary findings.

## 4 Materials and Methods

### 4.1 Plant Material

The aerial parts of *S. pinardii* were collected on April 21, 2023, from the entrance of Küllien Cave in the Geyikbayırı region of Konyaaltı (Antalya, Türkiye) at an altitude of 470 m (36°52′33″ N, 30°28′46″ E). Taxonomic identification of the plant was performed by Dr. Olcay Ceylan (Department of Biology, Muğla Sıtkı Koçman University, Muğla, Türkiye). A voucher specimen was deposited in the Herbarium of the Department of Biology, Muğla Sıtkı Koçman University, Muğla, Türkiye, under voucher number O.2388.

### 4.2 Extraction Procedure

The aerial parts of *S. pinardii* were air‐dried at room temperature in the shade for several weeks until a constant weight was reached. The dried plant material was then ground into small pieces using a laboratory blender. Particle size was not standardized or measured. For each extraction, 10 g of the powdered plant material was extracted with 200 mL of solvent (1:20, *w*/*v*). Ultrasound‐assisted extraction (UAE) was performed in a temperature‐controlled ultrasonic water bath operating at a frequency of 35 kHz and a nominal bath power of 320 W. The nominal power refers to the manufacturer's specified power output; the delivered acoustic power was not measured. The suspensions were sonicated for 60 min while maintaining the bath temperature below 35°C by periodically replacing the cooling water to minimize thermal degradation of phenolic compounds [31]. Following extraction, the mixtures were filtered through Whatman No. 1 filter paper. The aqueous extract was subsequently lyophilized, whereas the methanol extract was concentrated under reduced pressure using a rotary evaporator. The obtained extracts were stored at 4°C until further analysis. Extraction yields were 12.80% for the methanol extract and 19.70% for the aqueous extract. A single bulk extraction was performed for each solvent system, and aliquots obtained from these extracts were used for all subsequent phytochemical and biological analyses.

Methanol and water are widely employed solvents in plant extraction studies due to their effectiveness in recovering phenolic compounds with varying polarities. Methanol, as a moderately polar solvent, is particularly efficient in extracting a broad range of phenolics, including flavonoids and phenolic acids, whereas water is more suitable for highly polar compounds. The use of methanol and water as separate extraction solvents enables the recovery of compounds with different polarities by promoting solvent‐specific interactions with the plant matrix and improving the solubility of distinct phytochemical constituents. Furthermore, when coupled with ultrasound‐assisted extraction, cavitation effects facilitate the release of bioactive compounds from the plant matrix, resulting in improved yield and extraction performance [8].

### 4.3 Determination of Phenolic Composition

The TPC of the extracts was quantified using the Folin–Ciocalteu assay, and the results were expressed in terms of GAE. TFC, on the other hand, was determined employing the aluminum chloride colorimetric method, with findings reported as rutin equivalents (RE). Both analytical procedures were carried out based on the methods described by [32] with slight modifications [33, 34].

The detailed phytochemical profiling was performed using a previously validated LC–ESI–MS/MS analytical method [35]. Compound identification was achieved by comparison of retention times and optimized multiple reaction monitoring (MRM) transitions with those of authentic reference standards analyzed under identical chromatographic conditions. Representative LC–ESI–MS/MS product ion spectra together with the proposed fragmentation pathways of the four major phenolic constituents are provided in Figure S1. The corresponding analytical parameters are presented in Tables S1 and S2.

### 4.4 Biological Activity Assays

The antioxidant capacity of the extracts was comprehensively evaluated using a battery of in vitro assays, including the phosphomolybdenum total antioxidant capacity assay, 2,2‐diphenyl‐1‐picrylhydrazyl (DPPH) and 2,2′‐azinobis(3‐ethylbenzothiazoline‐6‐sulfonic acid) (ABTS) radical scavenging tests, ferrous ion chelation, cupric ion reducing antioxidant capacity (CUPRAC), and ferric reducing antioxidant power (FRAP), in accordance with previously reported methodologies [34, 36, 37, 38, 39], with slight modifications [40, 41].

For the phosphomolybdenum method, the extracts were incubated with a reagent mixture containing sulfuric acid, sodium phosphate, and ammonium molybdate at 95°C for 90 min, followed by absorbance measurement at 695 nm. DPPH radical scavenging activity was assessed by recording the decrease in absorbance of the DPPH methanol solution at 517 nm after reaction with the extracts under dark conditions. In the ABTS assay, the ABTS•^+^ radical cation was produced through the reaction of ABTS with potassium persulfate, and the reduction in absorbance was monitored at 734 nm.

Metal chelating activity was determined by measuring the formation of the ferrozine–Fe^2^^+^ complex at 562 nm. The reducing power of the extracts was further investigated using CUPRAC and FRAP assays, with absorbance values recorded at 450 and 593 nm, respectively.

The inhibitory effects of the extracts on key enzymes, namely α‐amylase, α‐glucosidase, tyrosinase, and cholinesterases (AChE and BChE), were evaluated based on established procedures [42, 43], with slight modifications [44]. α‐Amylase inhibition was determined using the Caraway–Somogyi iodine–potassium iodide method, while α‐glucosidase activity was measured using *p*‐nitrophenyl‐α‐D‐glucopyranoside (pNPG) as the substrate. Tyrosinase inhibition was assessed using L‐DOPA in a dopachrome‐based system. Cholinesterase inhibitory activity was evaluated by Ellman's colorimetric method using acetylthiocholine iodide or butyrylthiocholine chloride as substrates.

IC_50_ values for radical scavenging, metal chelation, and enzyme inhibition assays were calculated as the concentration required to achieve 50% inhibition. In contrast, EC_50_ values for phosphomolybdenum and reducing power assays were defined as the concentration corresponding to an absorbance of 0.500. All biological activity results were expressed as mg standard equivalents per g extract and compared with appropriate reference standards. Detailed methodological procedures are provided in the Supporting Information.

### 4.5 Statistical Analysis

All experiments were conducted in triplicate (*n* = 3), using aliquots obtained from a single bulk extraction prepared separately for each solvent, and the results are presented as mean ± standard deviation (SD). Thus, the triplicate measurements represent technical replicates rather than independent biological or extraction replicates. The absence of independently prepared replicate extractions is acknowledged as a limitation of the experimental design, as it restricts the assessment of variability associated with the extraction procedure. Statistical analyses were performed using IBM SPSS Statistics (IBM Corp., Armonk, NY, USA). For comparisons between two groups, the Student's *t*‐test was applied, whereas comparisons involving three or more groups were evaluated using one‐way analysis of variance (ANOVA) followed by Tukey's honestly significant difference (HSD) post hoc test. A value of *p* < 0.05 was considered statistically significant.

## Author Contributions

**Cengiz Sarikurkcu**: conceptualization, writing – original draft, writing – review and editing. **Arzuhan Sihoglu**: methodology and writing – review and editing. **Zeyneb Karakus**: material preparation, data collection and analysis, and writing – review and editing. The completed manuscript underwent comprehensive review by all authors, resulting in approval for publication. All authors have read and agreed to the published version of the manuscript.

## Ethics Statement

The authors have nothing to report.

## Plant‐Collection Compliance Statement

No specific permit was required for the collection of this plant material under the applicable local regulations. The identification of the plant material was performed by qualified taxonomists, and a voucher specimen has been deposited in a recognized herbarium for future reference.

## Conflicts of Interest

The authors declare no conflicts of interest.

## Use of Generative AI and AI‐Assisted Technologies in the Writing Process

Artificial intelligence (AI) tools were employed exclusively to support language refinement, improve conceptual clarity, and enhance the overall presentation of the manuscript. All experimental procedures, laboratory analyses, and data acquisition were conducted entirely by the authors, without any involvement of AI systems. The use of AI was limited to improving textual coherence, readability, and technical precision. All AI‐assisted modifications were critically evaluated and approved by the authors to ensure compliance with standards of scientific rigor, transparency, and research integrity.

## Supporting information




**Supporting File 1**: cbdv71812‐sup‐0001‐SuppMat.docx.

## Data Availability

The data that support the findings of this study are available from the corresponding author upon reasonable request.
